# Autophagy is involved in the replication of H9N2 influenza virus via the regulation of oxidative stress in alveolar epithelial cells

**DOI:** 10.1186/s12985-020-01484-x

**Published:** 2021-01-18

**Authors:** Rui-hua Zhang, Hong-liang Zhang, Pei-yao Li, Chun-hong Li, Jing-ping Gao, Jun Li, Tong Xu, Xue-jing Wang, Cun-lian Wang, Hui-chen Zhang, Ming-ju Xu, Shu-fei Tian

**Affiliations:** 1 Key Laboratory of Preventive Veterinary Medicine, Department of Veterinary Medicine, Animal Science College, Hebei North University, Zhangjiakou, 075131 Hebei People’s Republic of China; 2 Department of Veterinary Medicine, Inner Mongolia Agricultural University, Hohhot, 010018 Inner Mongolia People’s Republic of China; 3 Shandong Provincial Key Laboratory of Animal Disease Control and Breeding, Institute of Animal Science and Veterinary Medicine, Shandong Academy of Agricultural Sciences, Jinan, 250100 Shandong People’s Republic of China; 4 The Animal Husbandry and Veterinary Institute of Hebei, Baoding, 071001 Hebei People’s Republic of China; 5 He He Animal Husbandry Development Co. Ltd., Zhenlai, 137300 Jilin People’s Republic of China

**Keywords:** Autophagy, H9N2 influenza virus, Oxidative stress, Virus replication, Akt/mTOR

## Abstract

**Background:**

Oxidative stress is an important pathogenic factor in influenza A virus infection. It has been found that reactive oxygen species induced by the H9N2 influenza virus is associated with viral replication. However, the mechanisms involved remain to be elucidated.

**Methods:**

In this study, the role of autophagy was investigated in H9N2 influenza virus-induced oxidative stress and viral replication in A549 cells. Autophagy induced by H9N2 was inhibited by an autophagy inhibitor or RNA interference, the autophagy level, viral replication and the presence of oxidative stress were detected by western blot, TCID50 assay, and Real-time PCR. Then autophagy and oxidative stress were regulated, and viral replication was determined. At last, the Akt/TSC2/mTOR signaling pathways was detected by western blot.

**Results:**

Autophagy was induced by the H9N2 influenza virus and the inhibition of autophagy reduced the viral titer and the expression of nucleoprotein and matrix protein. The blockage of autophagy suppressed the H9N2 virus-induced increase in the presence of oxidative stress, as evidenced by decreased reactive oxygen species production and malonaldehyde generation, and increased superoxide dismutase 1 levels. The changes in the viral titer and NP mRNA level caused by the antioxidant, *N*-acetyl-cysteine (NAC), and the oxidizing agent, H_2_O_2_, confirmed the involvement of oxidative stress in the control of viral replication. NAC plus transfection with Atg5 siRNA significantly reduced the viral titer and oxidative stress compared with NAC treatment alone, which confirmed that autophagy was involved in the replication of H9N2 influenza virus by regulating oxidative stress. Our data also revealed that autophagy was induced by the H9N2 influenza virus through the Akt/TSC2/mTOR pathway. The activation of Akt or the inhibition of TSC2 suppressed the H9N2 virus-induced increase in the level of LC3-II, restored the decrease in the expression of phospho-pAkt, phospho-mTOR and phospho-pS6 caused by H9N2 infection, suppressed the H9N2-induced increase in the presence of oxidative stress, and resulted in a decrease in the viral titer.

**Conclusion:**

**A**utophagy is involved in H9N2 virus replication by regulating oxidative stress via the Akt/TSC2/mTOR signaling pathway. Thus, autophagy maybe a target which may be used to improve antiviral therapeutics.

## Background

Influenza A virus is a highly infective and pathogenic virus and causes seasonal epidemics and occasional global pandemics [1]. Although vaccines are used to prevent viral infection, knowledge of the pathogenic mechanisms is important, in order to develop treatment strategies for infected individuals. The major pathogenic factor of influenza A viral infection is the high viral loading and active viral replication [2]. Virus-induced hypercytokinemia is another pathogenic factor in influenza A virus infection [3]. Moreover, we previously demonstrated that oxidative stress triggered by H9N2 influenza A virus is involved in acute lung injury by regulating the Toll-like receptor 4 (TLR4) signaling pathway [4, 5] in mice. It has been reported that excess reactive oxygen species (ROS) production is generated in H9N2 influenza A virus-infected cells and mice, and results in significant damage to respiratory tissues [6]. The increase in ROS levels by IAV-induced superoxide dismutase 1 (SOD1) downregulation appears to influence viral replication [7]. The antioxidant treatment of influenza virus-infected cells has been shown to result in a lower viral titer, supporting an important role of oxidative stress in viral replication. However, the underlying mechanisms remain to be fully elucidated [8].

Autophagy is a catabolic process of unneeded proteins, damaged organelles and invading microbe degradation, and recycling that maintains intracellular homeostasis [9, 10]. Autophagy can be induced by pathogen infection and is triggered by the inhibition of mammalian target of rapamycin (mTOR) and Beclin1-phosphatidylinositol-3 kinase (PI3K) complex activation, followed by the initial formation of double-membraned vesicles known as the isolation membrane or phagophore [11]. Subsequently the phagophore elongates and encloses the cytoplasmic constituent to form the autophagosome. The elongation of the autophagosome requires Atg12-Atg5 and Atg8-PE (LC3-II in mammalian cells) ubiquitin-like conjugation systems. LC3-II attaches to the matured autophagosome membrane, thus serving as a marker for the formation of the autophagosome. Ultimately, autophagosomes fuse with lysosomes to form single-membrane autophagolysosomes where the autophagosome content is digested [12, 13].

Influenza A virus infection is known to induce autophagy in various host cells [14–18]. Influenza A virus relies on the acidification of the endosome-lysosome system to uncoat and release the ribonucleoproteins (RNPs) [19, 20], a situation which is also needed for lysosomal enzyme activity, autophagosome and lysosome fusion for the autophagy pathway [12]. Thus, autophagy may also be involved in influenza virus infection and replication [14, 17, 21–23]. It has been reported that autophagy can be induced by the H1N1, H3N2 and H9N2 influenza virus, and that it is involved in the replication and pathogenesis of influenza A virus [14, 16, 17, 22]. Autophagy is essential for the generation of proinflammatory cytokines and chemokines in influenza virus-infected human blood macrophages [22]. The administration of an autophagy inhibitor (evodiamine) has been shown to reduce the H1N1 influenza virus-induced death of A549 human lung epithelial cells [24]. These data indicate that autophagy is involved in the pathogenic mechanisms of influenza A virus. Although autophagy induced by the influenza virus has been reported to mediate cell death [25], the association between autophagy and oxidative stress remains unclear, and the role of autophagy in the pathogenic mechanisms of influenza A virus also remains unclear. It has been reported that influenza A virus infection induces ROS generation through SOD1 downregulation involving lysosomal proteolysis, and that lysosomes play a critical role in the cellular defense against invading pathogens through autophagic degradation [7]. Therefore, in this study, we investigated whether autophagy activated by the H9N2 influenza virus is involved in viral replication by regulating oxidative stress in alveolar epithelial cells. In addition, the signaling pathways involved were investigated to elucidate the underlying mechanisms.

## Materials and methods

### Viral strains and cell culture

The H9N2 influenza A/swine/HeBei/012/2008 virus (H9N2 virus) was isolated from pig lung tissues and stored in the Key Laboratory of Preventive Veterinary Medicine in Hebei North University. A549 human lung epithelial cell line cells were obtained from Nankai University, and maintained in DMEM medium with 10% heat-inactivated fetal bovine serum (FBS) in 5% CO_2_ at 37 °C.

### Cell treatments and virus infection

A549 cells were treated with 10 mM (final concentration) 3-MA, 10 μM (final concentration) LY294002 for 2 h, 100 nM (final concentration) rapamycin for 12 h, 10 ng/ml insulin-like growth factor 1 (IGF-1) for 24 h, followed by H9N2 virus infection (multiplicity of infection, MOI = 1) or an equal volume of the vehicle.

### Confocal imaging

A549 cells were grown on coverslips and transiently transfected with plasmids of EGFP-LC3 (Addgene) with Lipofectamine 2000 (Invitrogen; Thermo Fisher Scientific) based on the manufacturer’s instructions. At 24 h post transfection, the cells were treated with 3-MA or LY294002 for 2 h, followed by H9N2 virus infection (MOI = 1) for 24 h. The fluorescent images were recorded by use of a confocal laser scanning microscope (TCS SP5II, Leica, Ernst-Leitz-Strasse, Germany).

### RNA interference

A549 cells were plated and transiently transfected with 50 nM Atg5-specific siRNA, tuberous sclerosis 2 (TSC2)-specific siRNA or control siRNA using Lipofectamine 2000 (Invitrogen; Thermo Fisher Scientific) based on the manufacturer’s instructions. The siRNA control is the scramble siRNA and it has the same nucleotide composition as the target gene sequence. The sequences of Atg5-specific siRNA (Atg5 siRNA) and TSC2-siRNA were designed as previously described [25]. At 48 h post-transfection, the protein levels of Atg5 were detected by western blot analysis.

### Titration of viruses

The A549 cells were plated in 12-well plates and treated with 3-MA, LY294002, IGF-1, Atg5-specific siRNA, TSC2-specific siRNA or control siRNA, followed by the infection of H9N2 virus. Following 1 h of adsorption, the infected cells were washed with PBS and incubated at 37 °C for 36 h, the supernatants of the infected culture were collected at 12, 24 and 36 h post-infection (hpi) and serially diluted in serum-free DMEM. Ten-fold diluted culture was added to confluent cells in a 96-well plate for 1 hat 37 °C. Infected cells were washed with PBS and cultured with in DMEM with 2% FBS for 72 h. Viral titers were determined by 50% tissue culture infectious dose (TCID50) analysis on A549 cells using the Reed-Muench method.

### Detection of ROS, MDA and SOD1 levels

ROS production was measured using the total ROS/Superoxide Detection kit (Enzo Life Sciences Inc) according to the manufacturer's instructions. A549 cells were seeded in 6-well plates. After staining the cells with ROS/Superoxide detection reagent, the absorbance of the samples was analyzed using a multi-detection reader (Bio-Tek Instruments Inc.) at excitation wavelengths of 488 nm and emission wavelengths of 520 nm, respectively. ROS production was evaluated based on fluorescence intensity compared to the control cells. MDA and SOD1 levels in the A549 cells were detected using detection kits (Nanjing Jiancheng Institute of Biotechnology).

### Reverse transcription-quantitative PCR

Control siRNA-, Atg5 siRNA- and LY294002-transfected A549 cells were infected with the H9N2 influenza virus. Total RNA was extracted from the in cells using Trizol reagent (Invitrogen; Thermo Fisher Scientific) according to the manufacturer’s protocol. cDNA was synthesized from 1.5 μg of RNA with the cDNA Reverse Transcription kit (Apllied Biosystems). PCR amplification assays were performed with a SYBR Premix Ex Taq II kit (Takara) on an ABI 7300 Real-Time PCR system (Applied Biosystems). The expression of NP gene was normalized on the basis of the expression of β-actin. The primers used in this study are available upon request.

### Western blot analysis

A549 cells were collected, lysed and the protein concentration was determined using a BCA protein assay kit (ThermoFisher Scientific) according to the manufacturer’s protocol. Proteins from each sample were run on an SDS polyacrylamide gel. The protein bands were then transferred onto nitrocellulose filter membranes. The membranes were incubated with the specific antibodies against LC3B, Atg5, GAPDH, Akt, phosphorylated Akt, TSC2, phosphorylated TSC2, mTOR, phosphorylated mTOR, S6, phosphorylated S6, TSC2, NP, M2 (Cell Signaling Technology) and subsequently with horseradish peroxidase-labeled IgG secondary antibodies (Cell Signaling Technology). Finally, the membranes were visualized with an enhanced chemilumine scence system (Amersham Imager 600, GE, USA). The protein level was analysed by a densitometry using Alphalmager Series 2200 Software (Alpha Innotech, San Leandro, CA, USA). The relative protein level was normalized to GAPDH.

### Statistical analysis

Statistical analysis was performed with the SPSS statistical software package for Windows, version 18.0 (SPSS, Inc., Chicago, IL, USA). All data are presented as the means ± SD. Statistically significant differences among groups were calculated by one-way ANOVA followed by Tukey’s multiple comparisons test. P < 0.05 was considered significant.

## Results

### Autophagy is involved in H9N2 influenza virus replication

**Fig. 1:**
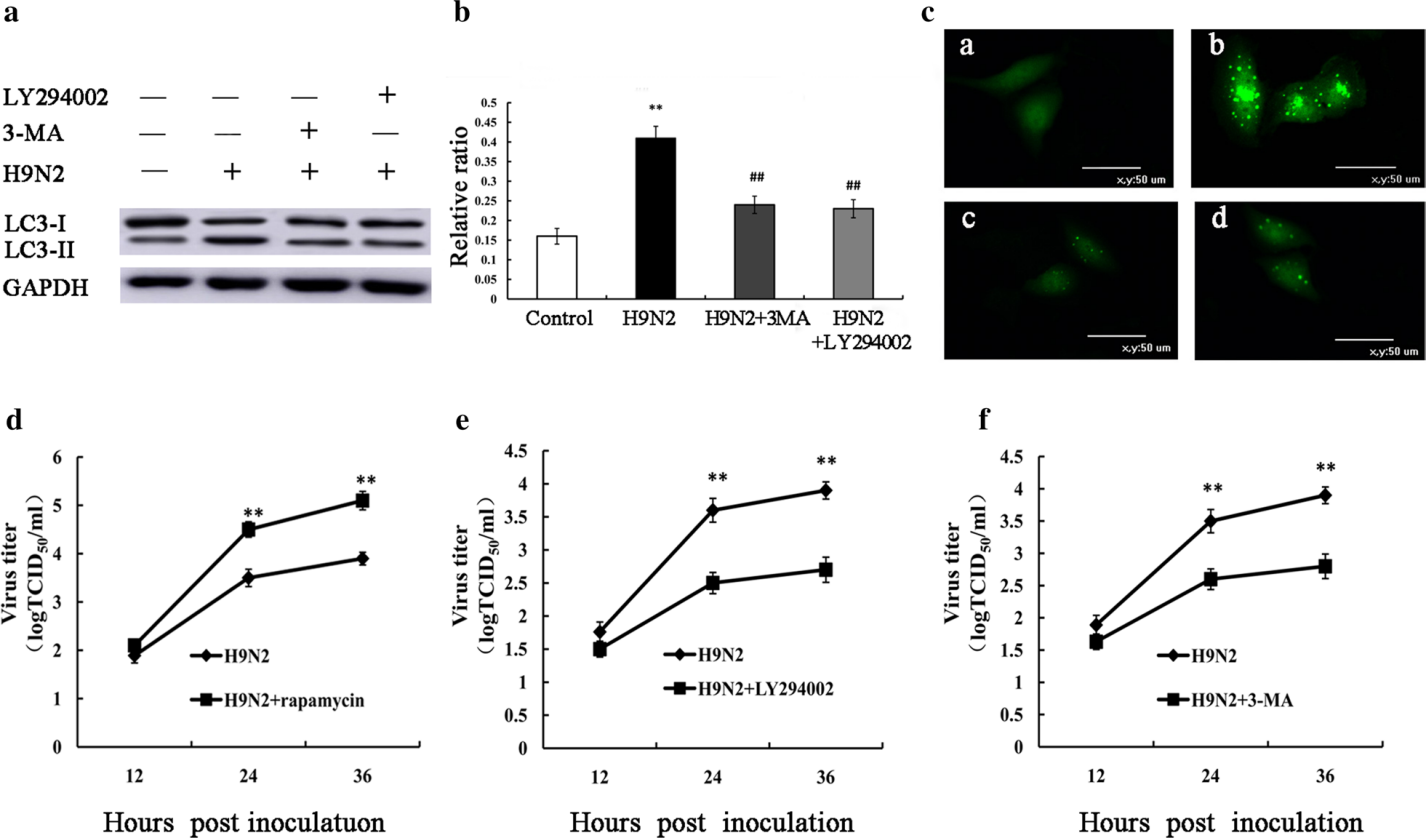
Autophagy is involved in H9N2 influenza virus replication (I). A549 cells were pre-treated with 10 μM LY294002, 10 mM 3-MA for 2 h, followed by infection with the H9N2 influenza virus (MOI = 1). (**a**, **b**) Cells were collected at 24 h p.i. and the protein expression levels of LC3-II were detected by western blot analysis. The expression levels of LC3-II were examined as ratios of intensities of GAPDH bands. **P < 0.01 relative to control. ^##^P < 0.01 relative to H9N2. (**c**) Fluorescence of GFP-LC3B was analyzed by confocal microscopy at 24 h p.i.. a: Control cells. b: H9N2 treated cells. c: H9N2 and LY294002 treated cells. d: H9N2 and 3-MA treated cells. (**d**–**f**) A549 cells were pre-treated with 100 nM rapamycin for 12 h, 10 μM LY294002, 10 mM 3-MA for 2 h, followed by infection with the H9N2 influenza virus. Supernatants from A549 cells were collected, and the viral titer was determined by measuring TCID_50_;**P < 0.01 relative to H9N2

**Fig. 2:**
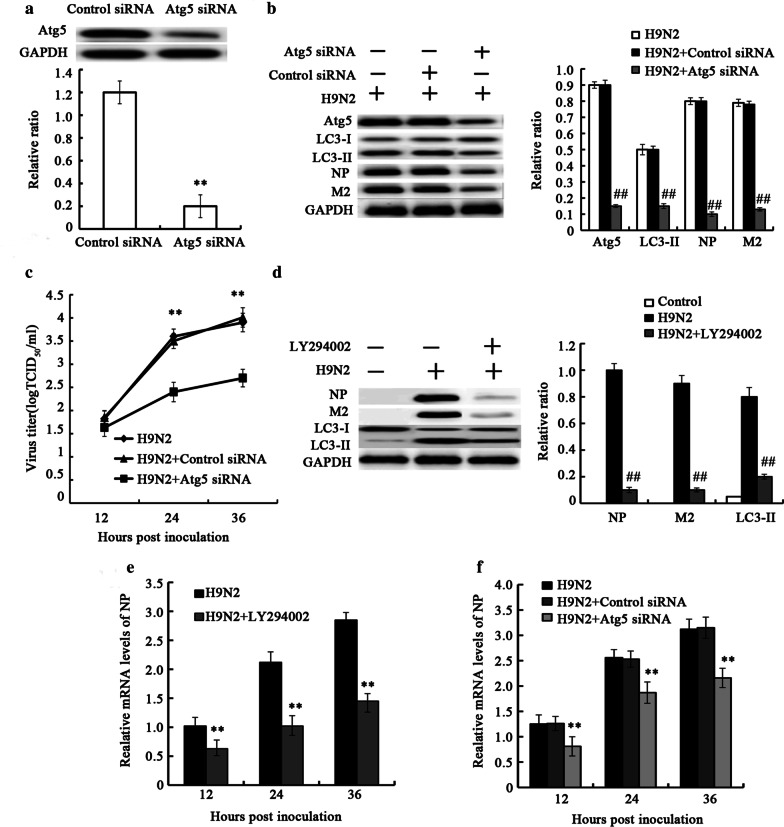
Autophagy is involved in H9N2 influenza virus replication (II). A549 cells were pre-treated with LY294002, control siRNA or Atg5 siRNA, followed by infection with the H9N2 influenza virus. The control siRNA is the scramble siRNA and it has the same nucleotide composition as the target gene sequence. (**a**) The protein expression levels of Atg5 were detected by western blot analysis. (**b**, **d**) Cells were collected and the protein expression levels of Atg5, LC3-II, NP, and M2 were detected by western blot analysis; **P < 0.01 relative to control, ^##^P < 0.01 relative to H9N2. (**c**) Supernatants from A549 cells were collected, and the viral titer was determined by measuring TCID_50_;**P < 0.01 relative to H9N2. (**e**, **f**) NP mRNA was detected by RT-PCR;**P < 0.01 relative to H9N2. The data are presented as the means ± SD (n = 5)

We first investigated the presence of H9N2 influenza virus-induced autophagy; thus, autophagosome formation was analyzed by detecting LC3-II expression and GFP-LC3 puncta. When autophagy is induced, LC3-I distribution in the cytoplasmis converted to LC3-II combined in the cell membrane by the addition of phosphatidylethanolamine to LC3-I [26, 27]. In this study, A549 cells were infected with H9N2 influenza virus, and the conversion of LC3-I to LC3-II was detected. The results revealed an increase in the level of LC3-II protein and GFP-LC3 puncta in the A549 cells infected with H9N2 virus (Fig. 1a–c), which demonstrated that the H9N2 influenza virus induced autophagy. Pretreatment with 3-MA and LY294002, the autophagy inhibitor, effectively attenuated the induction of autophagy by H9N2 virus (Fig. 1a–c). To evaluate the effect of autophagy on H9N2 virus replication, we applied autophagy activator, pharmacological inhibitor or RNA interference (Atg5 siRNA) to examine whether autophagy machinery was involved in H9N2 influenza virus replication. The A549 cells was treated with rapamycin, 3-MA, LY294002 and Atg5siRNA, followed by H9N2 influenza virus infection. The viral titer was then determined in supernatants of the cells by measuring TCID_50_. The results revealed that rapamycin treatment resulted in a significant increase in viral yield at 24 and 36 hpi (Fig. 1d). However, LY294002 treatment resulted in a significant decrease in viral yield (Fig. 1e). Similar to LY294002, 3-MA treatment markedly reduced the viral titer (Fig. 1f). To further investigate the effect of autophagy on H9N2 virus replication, the Atg5 gene required for the initiation of autophagy and which is involved in the Atg12–Atg5 ubiquitin-like conjugation systems mediating the elongation of the autophagosome [28], was knocked down prior to H9N2 influenza virus infection. The results of western blot analysis revealed that Atg5 was efficiently knocked down (Fig. 2a). Atg5 siRNA treatment resulted in a decrease in H9N2 virus-triggered autophagy, as indicated by the reduced level of LC3-II (Fig. 2b). The Atg5 siRNA-treated A549 cells infected with H9N2 influenza virus exhibited significantly decreased viral progeny titers, compared with the H9N2 influenza virus-infected cells treated with control siRNA (Fig. 2c).

To further evaluate the role of autophagy inH9N2 virus replication, autophagy was inhibited by LY294002 or Atg5 siRNA, and the expression of NP and M2 protein was detected. As shown in Fig. 2b, d, the expression levels of NP and M2 were significantly decreased in the cells treated with LY294002 or Atg5siRNA compared with the untreated cells. Simultaneously, LY294002 or Atg5 siRNA treatment significantly reduced the levels of viral NP mRNA (Fig. 2e, f). These results indicated that autophagy is essential for H9N2 influenza virus replication.

### Autophagy induced by H9N2 influenza virus is involved in the presence of oxidative stress

**Fig. 3:**
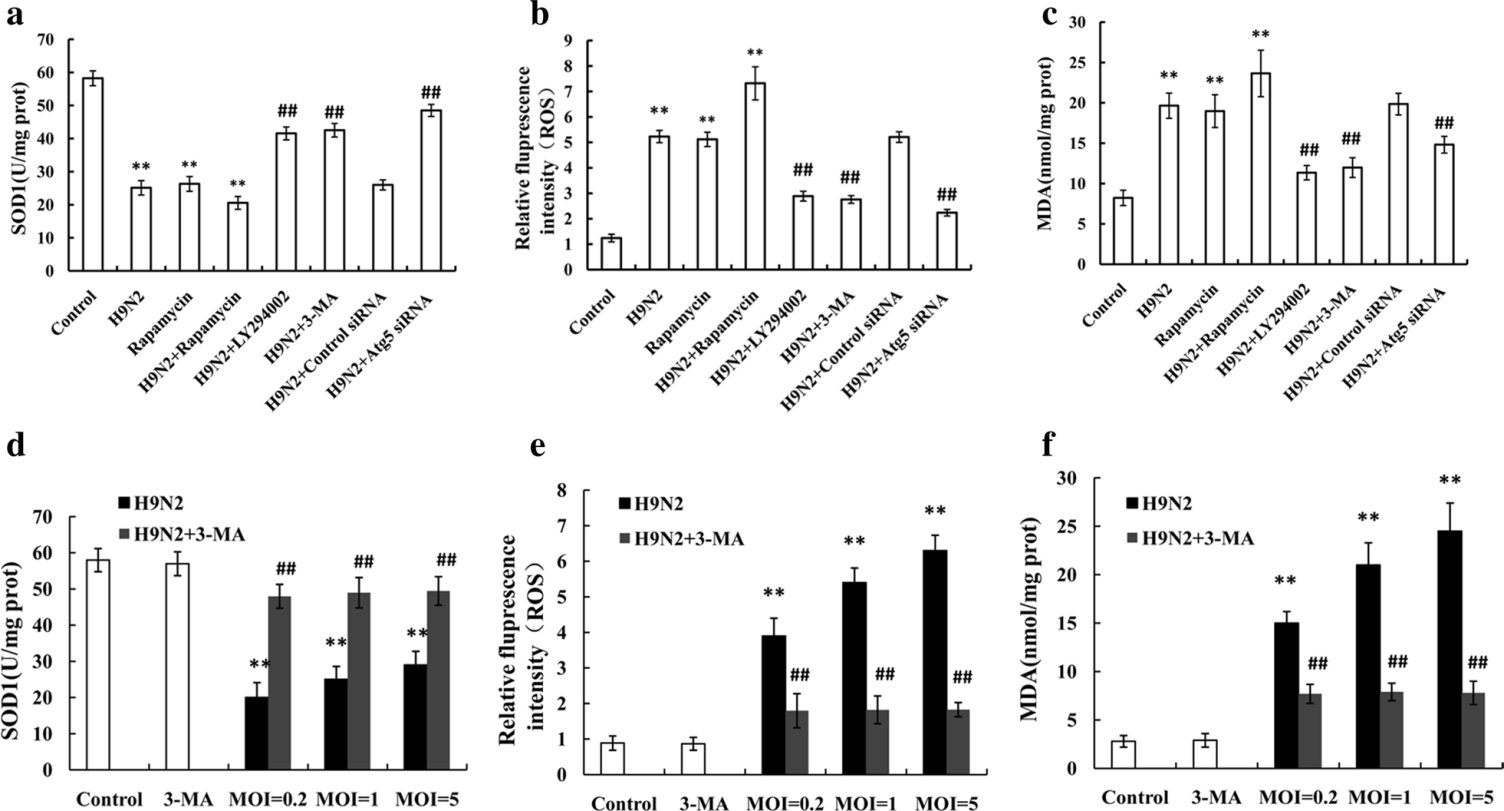
Autophagy induced by H9N2 influenza virus is involved in the presence of oxidative stress. A549 cells were pre-treated with rapamycin, LY294002, 3-MA, control siRNA or Atg5 siRNA, followed by infection with H9N2 influenza virus. (**a**) SOD1 activity, (**b**) ROS generation and (**c**) MDA contents were measured in the cells. A549 cells were pretreated with 3-MA, followed by infection with H9N2 influenza virus at an MOI of 0.2, 1 or 5. (**d**) SOD1 activity, (**e**) ROS generation and (**f**) MDA contents were measured in the cells. The data are presented as means ± SD (n = 5). **P < 0.01 relative to control, ^##^P < 0.01 relative to H9N2

Influenza A virus has been shown to alter the cellular ROS level by SOD1 downregulation [29]. Thus, in this study, we first investigated whether H9N2 influenza virus infection induces a decrease in SOD1 production. As shown in Fig. 3A, SOD1 activity in the A549 cells challenged with the H9N2 influenza virus was decreased compared with that in the unchallenged cells. A decrease in SOD1 levels can result in damaged redox balance in host cells; thus, we detected the production of ROS and MDA in H9N2 influenza virus-infected A549 cells. As shown in Fig. 3b, c, the production of ROS and MDA in the infected cells was significantly increased compared that in the uninfected cells. These data demonstrate that H9N2 influenza virus infection causes oxidative stress.

In order to determine whether autophagy is involved in oxidative stress induced by the H9N2 influenza virus, we used rapamycin, LY294002, 3-MA, or Atg5 siRNA to activate or inhibit autophagy prior to H9N2 influenza virus infection, and oxidative stress was detected. The results revealed that LY294002 treatment resulted in a decrease in the production of ROS and MDA compared that in the untreated cells (Fig. 3b, c). The LY294002-treated cells generated higher levels of SOD1 than the untreated cells (Fig. 3a). Similar results were observed in the 3-MA-treated cells (Fig. 3a–c). In addition, rapamycin treatment produced opposite results (Fig. 3a–c). To provide further evidence of the role of autophagy in oxidative stress induced by H9N2 influenza virus, we knocked down the autophagy-related gene, Atg5, by treating the cells with Atg5 siRNA. The results demonstrated that the A549 cells treated with Atg5 siRNA generated lower levels of ROS and MDA, as well as higher SOD1 levels than the control siRNA-treated cells (Fig. 3a–c).

As autophagy promotes to influenza virus replication, suppression of oxidative stress is possibly due to lower viral loads in the cells after blockage of autophagy. That is to say, virus itself but not autophagy directly contributes to oxidative stress. Hence, it is necessary to investigate if 3-MA suppresses oxidative stress equally after infection of different viral loads. A549 cells were pretreated with 3-MA, followed by H9N2 virus infection at MOI of 0.2, 1 or 5 at 24 h p.i., then the levels of oxidative stress was detected. The data showed that no significant difference in production of SOD1, ROS and MDA was found in 3-MA treated cells infected by H9N2 virus at an MOI of 0.2, 1, and 5 at 24 h p.i. (Fig. 3d–f). So, the results showed that autophagy inhibition, instead of lower viral loads after blockage of autophagy, was critical in the decreased oxidative stress in A549 cells. Therefore, this result confirmed that autophagy was closely associated with the oxidative stress induced by the H9N2 influenza virus.

### Autophagy-mediated oxidative stress influences the replication of H9N2 influenza virus

**Fig. 4:**
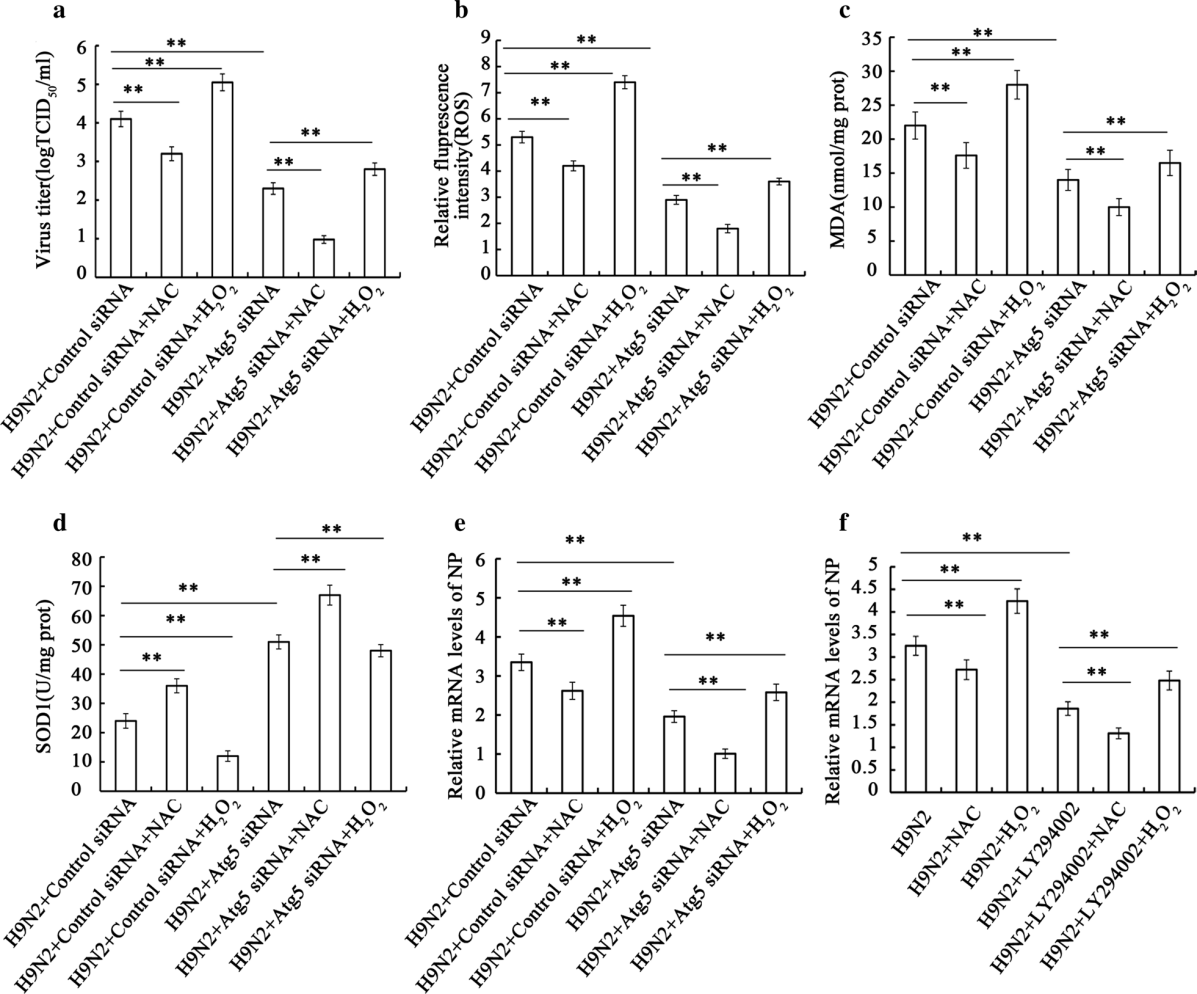
Autophagy-mediated oxidative stress influences the yield of H9N2 influenza virus. Following transfection with control siRNA or Atg5 siRNA, A549 cells were infected with the H9N2 influenza virus. (**a**) Following incubation for 24 h, the cells were treated with NAC (10 mM) or H_2_O_2_ (0.1 mM) for 6 h, supernatants were collected, and the viral titer was determined by measuring TCID_50_. (**b**) ROS generation, (**c**) MDA contents, and (**d**) SOD1 activity were measured in the cells. (**e**, **f**) A549 cells were also treated with Atg5 siRNA or LY294002, and NP mRNA was detected by RT-PCR. The data are presented as the means ± SD (n = 5). **P < 0.01

As described above, autophagy is involved in H9N2 influenza virus replication, since the inhibition of autophagy resulted in a decreased viral yield, as well as a decrease in the expression of NP and M2 proteins. Moreover, autophagy was found to be involved in the oxidative stress induced by the H9N2 influenza virus, as evidenced by the fact that the inhibition of autophagy led to reduced ROS and MDA generation, and increased SOD1 levels. Thus, we attempted to investigate whether oxidative stress regulated by autophagy influences viral replication. A549 cells were treated with or without Atg5 siRNA, followed by H9N2 influenza virus infection. The cells were then treated with NAC or hydrogen peroxide, and the viral titer, NP mRNA and the production of ROS, MDA and SOD1 were measured. The results revealed that combined treatment with NAC and Atg5 siRNA significantly reduced the viral titer (Fig. 4a) and was associated with decreased ROS and MDA generation, and increased SOD1 levels (Fig. 4b–d); of note, the effects of NAC were weaker than those of Atg5 siRNA. As was expected, hydrogen peroxide treatment produced opposite results (Fig. 4b–d). These results demonstrate that H9N2 influenza virus-induce doxidative stress regulated via the autophagy pathway benefits the replication of influenza.

To support the role of autophagy-mediated oxidative stress in the replication of the virus, we further employed Atg5 siRNA and LY294002 to inhibit autophagy, NAC or hydrogen peroxide to regulate oxidative stress, and the mRNA expression of NP gene was determined. As shown in Fig. 4e, Atg5 siRNA plus NAC treatment decreased the mRNA expression of NP, and the effects of NAC were also weaker than those of Atg5 siRNA. Hydrogen peroxide treatment also produced opposite results. Similar results were observed in the LY294002-treated A549 cells (Fig. 4f). Therefore, these data confirmed that H9N2 influenza virus-induced autophagy regulates oxidative stress, and that it thereby contributes to viral replication.

### Autophagy is involved in H9N2 influenza virus replication by regulating oxidative stress via the Akt/TSC2/mTOR signaling pathway

**Fig. 5:**
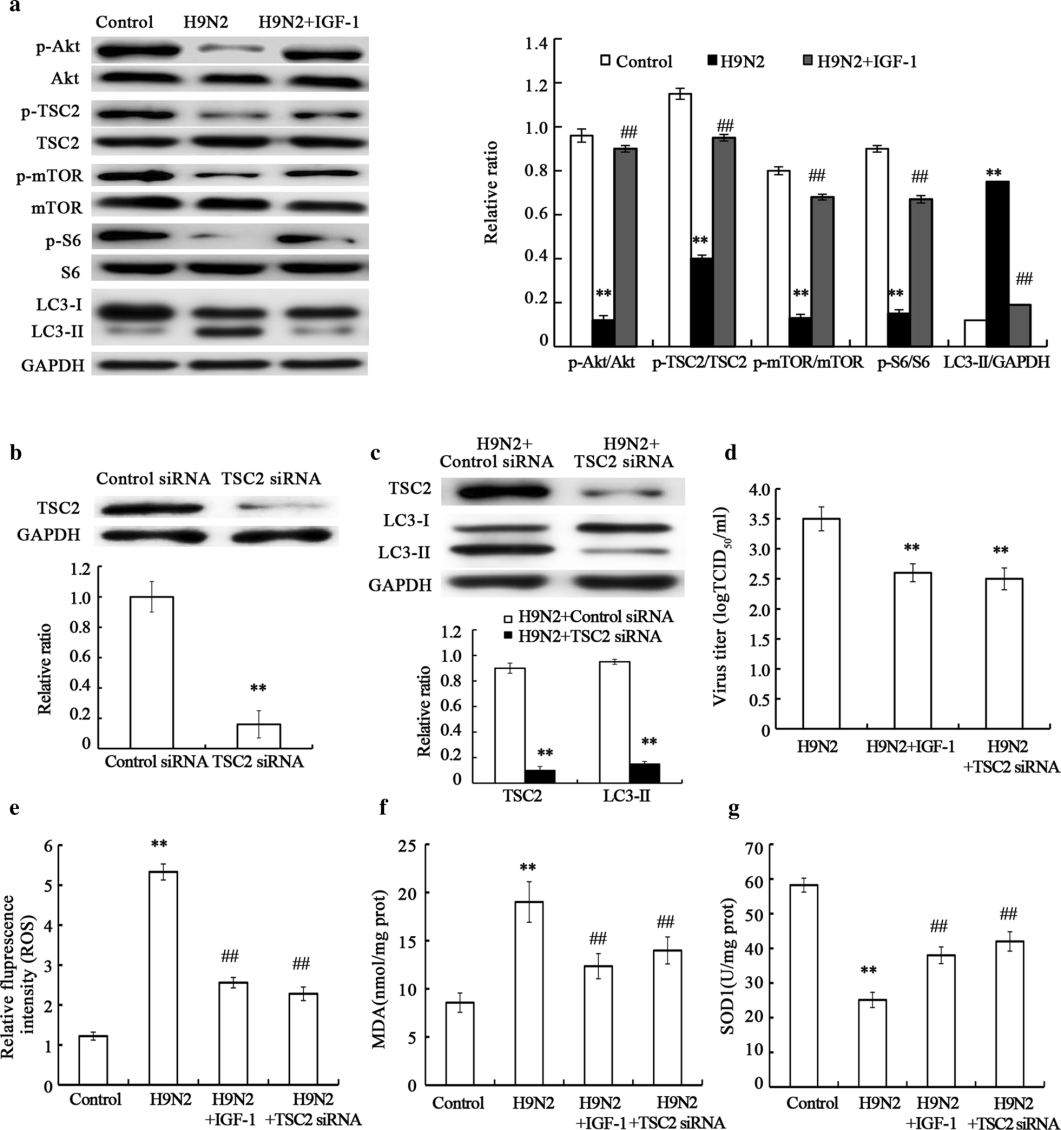
Autophagy is involved in H9N2 influenza virus replication by regulating the oxidative stress via Akt–TSC2–mTOR signaling pathway. A549 cells were pre-treated with IGF-1, followed by infection with H9N2 influenza virus. (**a**) Cells were collected and the protein expression levels of phospho-Akt, Akt, phospho-TSC2, TSC2, phospho-mTOR, mTOR, phospho-pS6, S6 and LC3-II were detected by western blot analysis. Following transfection with control siRNA or TSC2 siRNA, A549 cells were infected with the H9N2 influenza virus. (**b**, **c**) Protein expression levels of TSC2 and LC3-II were detected by western blot analysis. (**dd**) Supernatants from A549 cells were collected, and the viral titer was determined by measuring TCID_50_. (**e**) ROS generation, (**f**) MDA contents, and (**g**) SOD1 activity were measured in cells. The data are presented as the means ± SD (n = 5). **P < 0.01 relative to control, ^##^P < 0.01 relative to H9N2

Autophagy can be triggered by suppressing the mTOR signaling pathway [30, 31]. To investigate the mechanisms through which autophagy is involved inH9N2 influenza virus infection, we detected the Akt/TSC2/mTOR pathway by western blot analysis in the A549 cells. As shown in Fig. 4a, H9N2 influenza virus infection reduced the phosphorylation levels of AKT, TSC2 and mTOR in the A549 cells. Furthermore, the phosphorylation level of S6 (substrate of mTOR) in the A549 cells infected with theH9N2 influenza virus was significantly decreased. IGF-1, a potent stimulator of the PI3K/Akt pathway [32–34], was applied to further investigate the role of the Akt/mTOR pathway. The results revealed that IGF-1 treatment significantly restored the H9N2 influenza virus-induced decrease in the phosphorylation levels of AKT, TSC2, mTOR and S6. Moreover, treatment with IGF-1 significantly suppressed the H9N2 influenza virus-induced increase in the expression of LC3-II (Fig. 5a).

The PI3K/Akt/TSC2 pathway regulates mTOR-mediated autophagic signaling [35–37]. To further investigate whether H9N2 influenza virus-triggered autophagyis regulated by the Akt/TSC2/mTOR signaling pathway, the A549 cells weretransfected with TSC2siRNA, followed by H9N2 influenza virus infection. The results revealed that the TSC2 was efficiently knocked down (Fig. 5b) and that the production of LC3-II protein in the A549 cells treated withTSC2 siRNA was significantly decreased compared to that in the A549 cells treated with control siRNA (Fig. 5c), which confirmed TSC2 was involved in H9N2-induced autophagy. Taken together, these data demonstrated that H9N2 influenza virus induced autophagy by targeting the Akt/TSC2/mTOR signaling pathway.

To investigate whether autophagy induced by H9N2 influenza is involved in viral replication by regulating oxidative stress through the Akt/TSC2/mTOR signaling pathway, the A549 cells were treated with IGF-1 or TSC2 siRNA, followed by H9N2 influenza virus infection. We found that the viral titer was significantly decreased in the cells treated with TSC2 siRNA compared with the cells treated with control siRNA. IGF-1 treatment also resulted in a decrease in the viral titer (Fig. 5d). Furthermore, LC3-II expression was significantly reduced in the cells treated with IGF-1(Fig. 5a) or TSC2 siRNA (Fig. 5c). These data suggest that autophagy is involved in H9N2 influenza virus replication through the Akt/TSC2/mTOR signaling pathway. Moreover, we found that IGF-1 or TSC2 siRNA treatment significantly suppressed the H9N2 influenza virus-induced increase in the generation of ROS (Fig. 5e) and MDA (Fig. 5f), and restored the H9N2 influenza virus-induced decrease in the generation of SOD1(Fig. 5g). Taken together, these data demonstrate that autophagy is involved in H9N2 influenza virus replication by regulating oxidative stress via the Akt/TSC2/mTOR signaling pathway.

## Discussion

Influenza A virus is a worldwide and often lethal pathogen [38], and poses a serious threat to human health. It has been reported that the pathogenic factor of influenza A virus infection is the high viral loading and virus-induced hypercytokemia [2, 3]. However, the mechanisms responsible for the pathogenicity of the influenza virus have not been fully elucidated, which has stymied the development of antiviral treatment. In this study, we found that autophagy induced by the H9N2 influenza virus was involved in viral replication by regulating oxidative stress through the Akt/TSC2/mTOR signaling pathway in alveolar epithelial cells. Therefore, the activation of mTOR and/or the suppression of autophagy may prove to be effective therapeutic strategies with which to control the pathogenicity of the H9N2 influenza virus.

It has been reported that autophagy is actively involved in influenza A virus replication in A549 cells [14]. Influenza A virus is able to determine the death of its host cell by blocking macroautophagy; this has been shown in epithelial cell lines A549 [17]. The accumulation of autophagosomes has been observed in infected A549 human epithelial lung cells, and H5N1 has been shown to induce the autophagic death of A549 cells through mTOR [16]. A drug screening method has been established based on the autophagy pathway, which has been investigated in epithelial cell lines A549 [24]. These results indicate that A549 cells are an ideal cell line for studying autophagy induced by the influenza A virus. By contrast, BEASE-2B cells, a normal lung epithelial cell line, with similar epithelium-derived propriety and receptor pattern as A549 cells were unsusceptible to the infection and replication of influenza virus [39]. It has been reported that influenza virus replication was obviously poor in BEAS-2B cells in comparison with A549 cells, and the use of BEAS-2Bcells in influenza virus infection may not reflect the cytopathogenicity of influenza virus in human epithelial cells in vivo [40]. So, in this study, an adenocarcinoma cell line (A549) was used instead of a normal lung epithelial cell line such as BEAS-2B.

Influenza A virus M2 protein stimulates autophagosome formation in the early stage of viral infection [17]. Lipidated LC3-II can be detected at 3 h following influenza A virus infection in Ana-1 cells and at 6 h following infection inA549 cells [41]. It has been reported that autophagy is involved in the replication of influenza A virus [14]. In this study, we found that autophagy was induced by the H9N2 influenza A virus and that the inhibition of autophagy by autophagy inhibitor or siRNA significantly reduced the H9N2 influenza viral titer. Moreover, our research demonstrated that the blockage of autophagy resulted in a decrease in the mRNA expression of NP, and in the accumulation of NP and M2 protein; these findings are consistent with those of a previous study [42]. At the same time, the level of LC3-II has been shown to be markedly enhanced in cells transfected with NP and M2, and it has been suggested that NP and M2can induce autophagy [42]. Therefore, the expression of NP and M2 is related to autophagy. In short, autophagy is involved in the replication of the H9N2 influenza virus.

Viral diseases are related to physiological changes caused by the response of infected host cells. In this study, the association between autophagy and oxidative stress induced by influenza virus was investigated. The data demonstrated the autophagosomes induced by H9N2 influenza virus infection were one of the causes of the increase in cellular factors, such as ROS and MDA, and a decrease in cellular factors, such as SOD1. The knockdown of Atg5 or autophagy inhibitor suppressed the H9N2 virus-induced increase in the presence of oxidative stress, as evidenced by decreased ROS and MDA generation and increased SOD1 levels, which supported the important role of autophagy in influenza virus-induced redox control. Researchers have indicated that GSH results in a decrease in the viral titer of influenza A virus in lung homogenates of BALB/c mice [43]. H1N1 viral replication has been shown to be suppressed by carnosine treatment through the modulation of the respiratory burst and the generation of ROS in neutrophils [44]. The inhibition of ROS production suppresses viral ribonucleoprotein nuclear export and viral titer [45]. The viral titer of influenza A virus has been shown to be decreased by treatment with tje Nox2 inhibitor, apocynin [46]. Nox2 is the catalytic subunit of the phagocyte NADPH oxidase (NOX) involved in phagocytic ROS production [47]. Viral replication has been reported to be regulated by the redox state of the host cell [48]. The findings of this study demonstrated that the blockage of autophagy by Atg5siRNA significantly reduced the viral replication and suppressed the increase in the generation of ROS and MDA caused by the H9N2 virus, and also restored the decrease in SOD1 production induced by the virus, indicating that the cellular redox status mediated by autophagy plays an important role in viral replication. We also confirmed the importance of the redox state regulated by autophagy in viral replication by combined treatment with the antioxidant, NAC, and Atg5 siRNA following H9N2 influenza virus infection. Thus, autophagy is involved in H9N2 virus replication by influencing the redox state of A549 cells.

It has been reported that AKT, serine–threonine kinase, plays important roles in autophagy. Akt phosphorylates TSC2, and TSC2 is a GTPase-activating protein that can stimulate the GTPase activity of the GTPase, Rheb [48, 49]. Rheb, in its GTP-bound form, is an activator of mTOR. The TSC1/TSC2 complex inhibits mTOR signaling, and the phosphorylation of TSC2 reverses this inhibition. mTOR then inhibits the ULK1/2 complex, a complex required in the early stages of autophagy [50, 51], thus decreasing the level of autophagy [52, 53]. In a word, autophagy can be induced via the downregulation of the AKT/TSC2/mTOR pathway. H5N1 infection has been shown to result in autophagic cell death through the suppression of mTOR signaling and the inhibition of autophagy reduces H5N1-induced cell death [25]; this indicates that autophagy can be triggered by suppressing the mTOR signaling pathway [54]. The influenza virus has been reported to increase the accumulation of viral elements during viral replication by regulating autophagy via the mTOR/p70S6K signaling pathway [55]. The spectrum of genes in A549 cells infected with influenza A virus has been obtained and genes have been identified in the mTOR pathway [56]. In this study, autophagy benefited the replication of influenza through the Akt/TSC2/mTOR signaling pathways. Moreover, the activation of Akt or the inhibition of the TSC1/TSC2 complex suppressed the H9N2 virus-induced increase in the level of LC3-II and the presence of oxidative stress, and resulted in a decrease in the viral titer. Taken together, the findings of this study demonstrated autophagy is involved in H9N2 influenza viral replication by regulating oxidative stress through the Akt/TSC2/mTOR signaling pathway.

## Conclusion

This study demonstrates that autophagy is involved in the replication of H9N2 influenza virus by regulating oxidative stress through the Akt/TSC2/mTOR pathway in alveolar epithelial cells. This study also demonstrates the association between H9N2-triggered autophagy and oxidative stress, and demonstrates the mechanisms through viruses utilize the host defense system for its own replication, which enriches the pathogenesis of the H9N2 virus. The activation of mTOR and/or the suppression of autophagy may be used in the treatment of influenza A virus infection.

## Data Availability

Data are available on reasonable request.

## Abbreviations

IAV: influenza A virus
ROS: reactive oxygen species
PMVECs: pulmonary microvascular endothelial cells
3-MA: 3-methyladenine
NP: nucleoprotein
M2: matrix protein
MDA: malonaldehyde
SOD1: superoxide dismutase 1
NAC: *N*-acetyl-cysteine
H_2_O_2_: hydrogen peroxide
Atg5: autophagy-related 5
TSC2: tuberous sclerosis 2
mTOR: mammalian target of rapamycin
IGF-1: insulin-like growth factor-1
LC3-II: lapidated microtubule-associated protein 1 light chain 3
